# Glutamate attenuates lipopolysaccharide induced intestinal barrier injury by regulating corticotropin-releasing factor pathway in weaned pigs

**DOI:** 10.5713/ab.21.0476

**Published:** 2022-03-01

**Authors:** Junjie Guo, Tianzeng Liang, Huifu Chen, Xiangen Li, Xiaorui Ren, Xiuying Wang, Kan Xiao, Jiangchao Zhao, Huiling Zhu, Yulan Liu

**Affiliations:** 1 Hubei Key Laboratory of Animal Nutrition and Feed Science, Hubei Collaborative Innovation Center for Animal Nutrition and Feed Safety, Wuhan Polytechnic University, Wuhan430023, China; 2 Department of Animal Science, Division of Agriculture, University of Arkansas, Fayetteville, AR 72701, USA

**Keywords:** Corticotrophin-releasing Factor (CRF) Signaling Pathway, Glutamate, Intestinal Barrier Function, Lipopolysaccharide, Weaned Pig

## Abstract

**Objective:**

The purpose of this study was to evaluate the protection of glutamate (GLU) against the impairment in intestinal barrier function induced by lipopolysaccharide (LPS) stress in weaned pigs.

**Methods:**

Twenty-four weaned pigs were divided into four treatments containing: i) non-challenged control, ii) LPS-challenged control, iii) LPS+1.0% GLU, and iv) LPS+2.0% GLU. On day 28, pigs were treated with LPS or saline. Blood samples were collected at 0, 2, and 4 h post-injection. After blood samples collection at 4 h, all pigs were slaughtered, and spleen, mesenteric lymph nodes, liver and intestinal samples were obtained.

**Results:**

Dietary GLU supplementation inhibited the LPS-induced oxidative stress in pigs, as demonstrated by reduced malondialdehyde level and increased glutathione level in jejunum. Diets supplemented with GLU enhanced villus height, villus height/crypt depth and claudin-1 expression, attenuated intestinal histology and ultrastructure impairment induced by LPS. Moreover, GLU supplementation reversed intestinal intraepithelial lymphocyte number decrease and mast cell number increase induced by LPS stress. GLU reduced serum cortisol concentration at 4 h after LPS stress and downregulated the mRNA expression of intestinal corticotropin-releasing factor signal (corticotrophin-releasing factor [*CRF*], CRF receptor 1 [*CRFR1*], glucocorticoid receptor, tryptase, nerve growth factor, tyrosine kinase receptor A), and prevented mast cell activation. GLU upregulated the mRNA expression of intestinal transforming growth factor β.

**Conclusion:**

These findings indicate that GLU attenuates LPS-induced intestinal mucosal barrier injury, which is associated with modulating CRF signaling pathway.

## INTRODUCTION

Intestinal mucosa is not only the pivotal part for digestion and absorption of nutrients, but also the critical barrier between the luminal contents and systemic circulation. Intactness of intestinal mucosal barrier is crucial to the health and welfare of animals. Disturbance of intestinal mucosal barrier function can result in an increase of mucosal permeability, which is implicated in pathogenesis of multiple gastrointestinal diseases such as inflammatory bowel disease, necrotizing enterocolitis, and multiple organ system dysfunction [1].

It is well known that physical, psychological, and chemical stress can impair barrier function and integrity of intestine. The mechanisms of stress-induced intestinal injury involve corticotrophin-releasing factor (CRF) and mast cells [2]. A typical stress response is to activate the hypothalamic-pituitary-adrenal (HPA) axis resulting in CRF and adrenal glucocorticoids release [3]. Previous studies have confirmed that CRF plays its role by activating mast cell [2]. After activation, intestinal mucosal mast cells release mediators, like proteases and tumor necrosis factor alpha, which impair intestinal barrier function and induce intestinal inflammation [2].

After damage to the intestinal barrier, rapid resealing of the intestinal barrier is critical to preserve homeostasis. The continuity of the surface epithelium is reestablished by three processes. The healing processes begins with migration of intestinal epithelial cells to the injured site. This process is called epithelial renovation [4]. After renovation, epithelial cell begins to proliferate, and differentiate. These three wound-healing processes are regulated by various molecular and cellular signaling pathways. Studies have shown that transforming growth factor β (TGF-β) pathway plays a key role in intestinal mucosal wound repair [5].

Several studies have shown that some nutrients or non- peptide factors such as polyamines, short-chain fatty acids and glutamine can modulate intestinal epithelial wound healing, and maintain barrier function [6,7]. Glutamate (GLU) is classified as a nutritionally non-essential amino acid. GLU is also a multifunction amino acid involved in cell metabolism and physiology. GLU can promote the proliferation of cells outside the gastrointestinal tract [8]. Moreover, GLU serves as a precursor of the amino acid glutamine. GLU and glutamine are fundamental energy substrates for the small intestine. Dietary supplementation with GLU restored mucous circulation and prevented the apoptosis of enterocytes [9]. After weaning, the supply of dietary GLU in the gut of piglets is limited due to significant reduction of food intake, which is related to severe intestinal atrophy, inflammation, malabsorption, and death [10].

For the present study, we hypothesized that GLU could preserve intestinal integrity and barrier function through modulating CRF and TGF-β signaling pathway. We established the model of acute intestinal damage in weaned piglets by injecting *Escherichia coli* lipopolysaccharide (LPS). The effects of LPS are attributed to a cascade of pro-inflammatory cytokine synthesis and release. Overproduction of pro-inflammatory cytokines leads to a breakdown in intestinal integrity and epithelial function [11]. Therefore, LPS is commonly used to induce acute stress and study the effects of dietary regimes [11]. In addition, pigs are used as an experimental model for humans, because they are considered as a reliable model to study digestive physiology [12]. The aim of the study was to analyze whether dietary GLU supplementation could mitigate the damage of intestinal barrier function induced by LPS through modulation of CRF and TGF-β signaling pathway.

## MATERIALS AND METHODS

### Animals, diets and experimental design

Animal experiment was conducted in compliance with the Animal Scientific Procedures Act 1986 (Home Office Code of Practice. HMSO: London January 1997). Experimental procedures were approved by the Animal Care and Use Committee of Wuhan Polytechnic University, Hubei Province, China (EM423). A total of 24 pigs (Duroc×Large White× Landrace, 28±2 d of age, initial body weight (BW) = 7.02± 0.21 kg) were used in this experiment. According to the litter of origin and BW, pigs were randomly assigned to four treatment groups. Each group included 6 pigs (replicates) and each pig was in its own pen (cage). The pens were stainless steel pens with size 1.8×1.10 m. During the whole experiment, the pigs were in good health and the living environment was consistent with animal welfare guidelines. Water and experimental diets were freely available. Ingredient composition of basal diets were reported previously [13].

The treatments included: i) non-challenged control (CONTR, pigs fed with control diet and injected with sterile saline); ii) LPS-challenged control (LPS, pigs fed with control diet and injected with *Eschericha coli* LPS, [*Escherichia coli* serotype O55: B5, Sigma Chemical Inc., St. Louis, MO, USA]); iii) LPS+1.0% GLU treatment (pigs fed with 1.0% GLU diet and injected with *Eschericha coli* LPS); iv) LPS+2.0% GLU treatment (pigs fed with 2.0% GLU diet and injected with *Eschericha coli* LPS). The doses of GLU (L-GLU; purity >99%; Amino Acid Bio-Chemical Company Limited, Wuhan, China) were selected based on our previous study [13]. All diets were maintained isonitrogenous by supplementation of 1.21%, 0.61%, and 0% alanine (L-alanine, purity>99%, Amino Acid Bio-Chemical Company Limited, China) to the control, 1.0% GLU, and 2.0% GLU diets. The BW and feed intakes of pigs were recorded on d 1 and 28.

On d 28 of the experiment, pigs in 1.0% GLU, 2.0% GLU and challenge group were injected intraperitoneally with *Escherichia coli* LPS at 100 μg/kg BW and pigs in the unchallenged group were injected with the same amount of 0.9% (wt/vol) saline. The dosage of LPS used in the present study was based on our previous experiment [13]. The LPS was dissolved in sterile 0.9% salt solution.

### Sample collection

Before blood collection, sedatives were given to pigs to reduce stress during sampling. All pigs were bled via anterior vena cava at 0, 2, and 4 h after treatment of LPS or sterile saline. Serum was separated by centrifugation and stored −80°C for later analysis of cortisol and amino acids. After blood samples harvest at 4 h, all pigs were slaughtered by overdose sodium pentobarbital. Spleen, mesenteric lymph nodes (MLN) and liver were obtained under aseptic condition for measuring bacterial translocation. The small intestine was removed, and gently cleaned with phosphate buffered saline (PBS). Mid-jejunum and ileum (2 to 3 cm) were collected for a microscopic measurement of the mucosal morphology and transmission electron microscopy examination. About 12-cm segments from the middle of jejunum and ileum were opened longitudinally and cleaned with PBS. Mucosal samples were obtained by scraping with a glass slide, and snap-frozen in liquid nitrogen, then stored at −80°C.

### Serum concentrations of amino acids and cortisol

Eighteen amino acids in serum (4 h after administration of LPS) were analyzed by reverse-phase high performance liquid chromatography (HPLC) after derivatization with *o*-phthaldialdehyde. The amino acids included arginine, histidine, isoleucine, leucine, lysine, phenylalanine, methionine, threonine, tryptophan, valine, glycine, serine, tyrosine, asparagine, aspartic acid, GLU, glutamine, and alanine. Briefly, serum samples were acidified with 1.5 M HClO_4_, then neutralized with 2 M K_2_CO_3_. The extract was directly used for the analysis of amino acid by HPLC.

Serum concentrations of cortisol were determined using commercial ^125^I RIA assay kits (Bejing North Institute of Biological Technology, Beijing, China) according to the manufacturer’s protocols. The detectable ranges were 10 to 500 ng/mL. The sensitivity of the kit was 2 ng/mL, and the intra-assay coefficient of variation was <10%.

### Intestinal mucosal malondialdehyde and glutathione concentration

According to the manufacturer’s instructions (Nanjing Jiancheng Biotechnology Institute, Nanjing, China), the concentrations of malondialdehyde (MDA) and glutathione (GSH) in intestinal mucosa were measured using a commercial MDA and GSH kits. The protein concentrations of intestinal mucosa were quantified using the Coomassie Brilliant Blue G-250.

### Bacterial translocation

Bacterial translocation in the intestinal tract was assessed according to the method of Yang et al [14]. In short, the samples of MLN, spleen and liver were weighed respectively, and homogenized in 10 volumes of ice-cold sterile 0.9% saline. Tissue homogenates (50 μL) from different organs were evenly coated on blood and MacConkey’s agar plates and cultured at 37°C for 24 h under aerobic conditions. After incubation, the colonies were counted by a microbiologist who was unaware of the experimental design. The results were expressed as log 10 colony-forming units (CFU)/g of tissue’s weight.

### Small intestinal morphology

Mid-jejunum and ileum fixed with 4% paraformaldehyde-phosphate buffer were embedded with Paraffin wax, sectioned (5 μm) and stained with hematoxylin and eosin. Villus height, crypt depth and the number of intraepithelial lymphocytes (IEL) and goblet cells were measured.

Neutrophils were counted in accordance with cellular and nuclear morphology. For quantification of mast cells, jejunum and ileum were fixed in Carnoy’s fluid and sectioned (5 μm) for toluidine blue staining. Cell counts were conducted at ×40 magnification using the image analysis program on six different fields per slide and 6 slides per treatment group and expressed as number of cells/mm^2^. Cell counts and intestinal morphologic determination were conducted using a blind protocol.

### Transmission electron microscopy examination

Jejunal and ileal samples from each pig (6 pigs per group) were fixed with 2.5% glutaraldehyde and postfixed in 1% osmium tetroxide. Samples were then dehydrated in graded acetones, embedded in Epon 812 (Eimicon, Shanghai, China). Ultra-thin sections were cut and stained with uranyl acetate and lead citrate. Ultrastructural observations of the intestine were performed using a transmission electron microscope (Tecnai, FEI, Hillsboro, OR, USA) at an accelerating voltage of 200 kV and a magnification of 5,000 in blind manner.

### Western blotting analysis

Intestinal tissue samples (100 to 150 mg, jejunum, and ileum) from each pig (6 pigs per group) were homogenized separately in cold lysis buffer, then were centrifuged to remove insoluble material. Proteins of intestinal mucosa were loaded onto 10% separation gel for electrophoresis and transferred to polyvinylidene difluoride membranes. Non-specific binding in membranes were blocked with 3% bovine serum albumin in Tris-buffered saline (TBS)-Tween-20 buffer for 1 h at 21°C to 25°C. Rabbit anti-human claudin-1 antibody (1:1,000; Invitrogen Technology, Danvers, MA, USA) or mouse anti-β-actin antibody (monoclonal; 1:10,000; Sigma Aldrich, St. Louis, MO, USA) was added, incubated at 4°C overnight (12 to 16 h). The membranes were subsequently incubated with goat anti-rabbit or mouse immunoglobulin G horseradish peroxidase conjugated secondary antibody (1:5,000; AntGene Biotech Co., Ltd, Wuhan, China) for 2 h at room temperature. Specific bands were visualized with enhanced Chemiluminescence Western blotting kit (Amersham Biosciences, Uppsala, Sweden). The bands’ density were quantified using GeneTools software (Syngene, Frederick, MD, USA).

### mRNAexpression analysis by real-time quantitativepolymerase chain reaction

Total RNA was extracted from the small intestine, cDNA synthesis and quantitative real-time polymerase chain reaction (PCR) was performed. The primer sequences, accession numbers for the target and housekeeping genes (*CRF*, CRF receptor 1 [*CRFR1*], CRF receptor 2 [*CRFR2*], glucocorticoid receptor [*GR*], Tryptase, nerve growth factor [*NGF*], tyrosine kinase receptor A [*TrkA*], *TGF-β1*, epidermal growth factor receptor [*EGFR*] and glyceraldehyde-3-phosphate dehydrogenase [*GAPDH*]) are presented in Table 1. The expression of the target genes relative to housekeeping gene (*GAPDH*) was analyzed by the 2^−ΔΔCT^ method [15]. Relative mRNA abundance of each target gene was normalized to the control group.

### Statistical analyses

Data were analyzed by analysis of variance (ANOVA) using the general linear model procedure of SAS (SAS Inst. Inc., Cary, NC, USA). One pig was the experimental unit. Duncan’s multiple range tests were used to determine differences between treatments following ANOVA. Probability values less than 0.05 were used as the criterion for statistical significance.

## RESULTS

### Growth performance

During days 1 to 27 of the experiment, there was no significant differences of average daily gain, average daily feed intake, and feed/gain among four treatments (Table 2).

### Intestinal mucosal malondialdehyde and glutathione concentration

**Figure 1:**
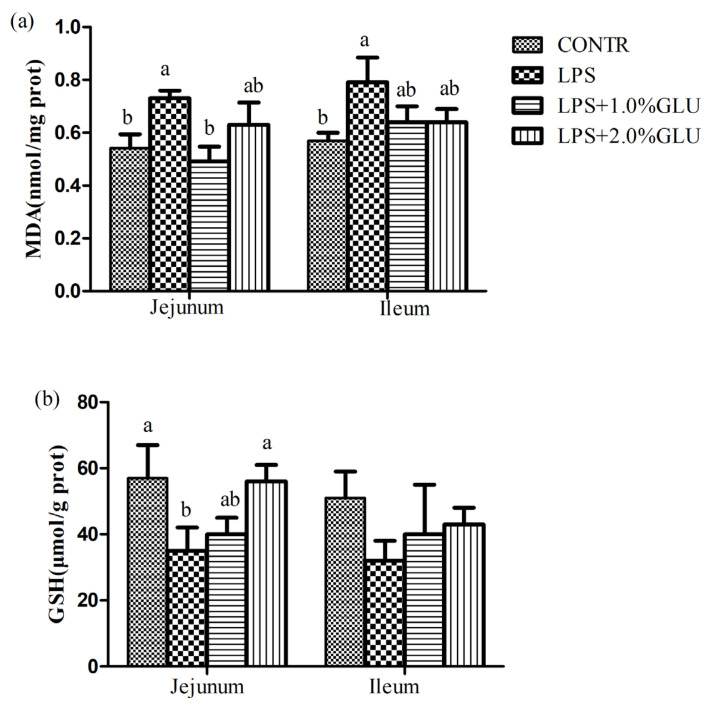
Effects of dietary glutamate (GLU) on intestinal (a) malondialdehyde (MDA) and (b) glutathione (GSH) concentrations in weaned pigs challenged by *Escherichia coli* lipopolysaccharide (LPS). Values are means and standard error of the mean, n = 6 (1 pig/pen). ( 
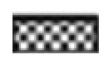
) Pigs fed a control diet and injected with sterile saline (CONTR); ( 
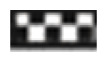
) pigs fed control diet and injected with LPS (LPS); ( 
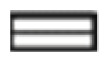
) pigs fed a 1.0% GLU diet and injected with LPS (LPS+1.0% GLU); ( 
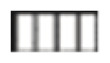
) pigs fed a 2.0% GLU diet and injected with LPS (LPS+2.0% GLU). ^a,b^ Bars with different letters indicate a significant difference (p<0.05).

LPS-challenged pigs had higher MDA concentration in jejunum (p = 0.044) and ileum (p = 0.025) compare with CONTR pigs (Figure 1). Dietary supplementation with 1.0% GLU decreased MDA concentrations in jejunum (p = 0.013) compared with LPS-challenged pigs. LPS-challenged pigs had lower GSH levels in jejunum (p = 0.036) compared with pigs in CONTR group. Dietary supplementation with 1.0% GLU enhanced jejunal GSH levels (p = 0.044) compared with pigs challenged with LPS.

### Bacterial translocation

LPS-challenged pigs had higher translocation microorganisms in spleen (p = 0.041) and tended to have higher translocation microorganisms in liver (p = 0.098) compared with pigs in CONTR group (Table 3). However, GLU supplementation did not affect translocation microorganisms of spleen and liver. Moreover, there was no significant difference in translocation microorganisms of MLN among all groups.

### Intestinal histomorphology

**Figure 2:**
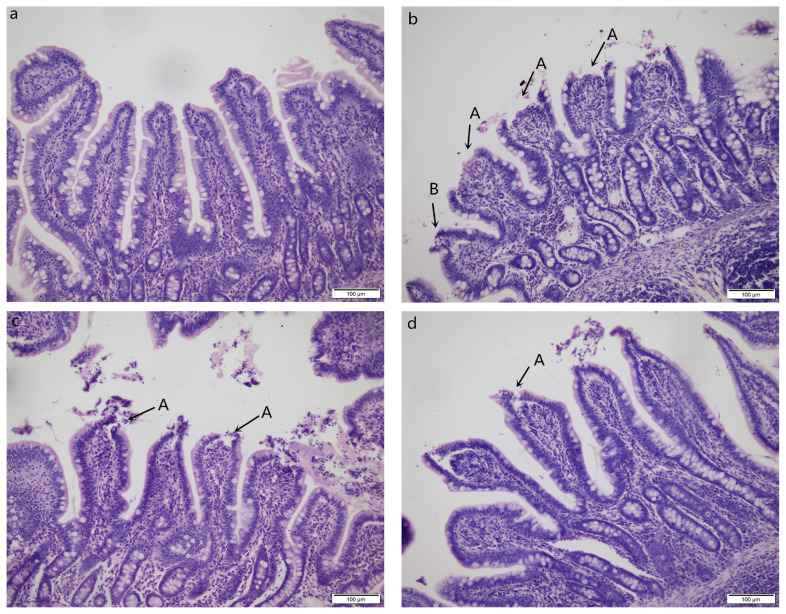
Intestinal mucosal morphology of jejunum (hematoxylin and eosin stained). (a) Pigs fed a control diet and injected with sterile saline (CONTR); No obvious changes were found. (b) Pigs fed control diet and injected with lipopolysaccharide (LPS); Intestinal mucosa was damaged by LPS. Arrows indicate the shedding of epithelium at the tip of the villus (A) and villous atrophy (B). (c) Pigs fed a 1.0% GLU diet and injected with LPS (LPS+1.0% GLU); Intestinal mucosal damage was alleviated. (d) Pigs fed a 2.0% GLU diet and injected with LPS (LPS+2.0% GLU). Intestinal mucosal injury was significantly attenuated. Original magnifications 200×. Scale bars = 100 μm.

**Figure 3:**
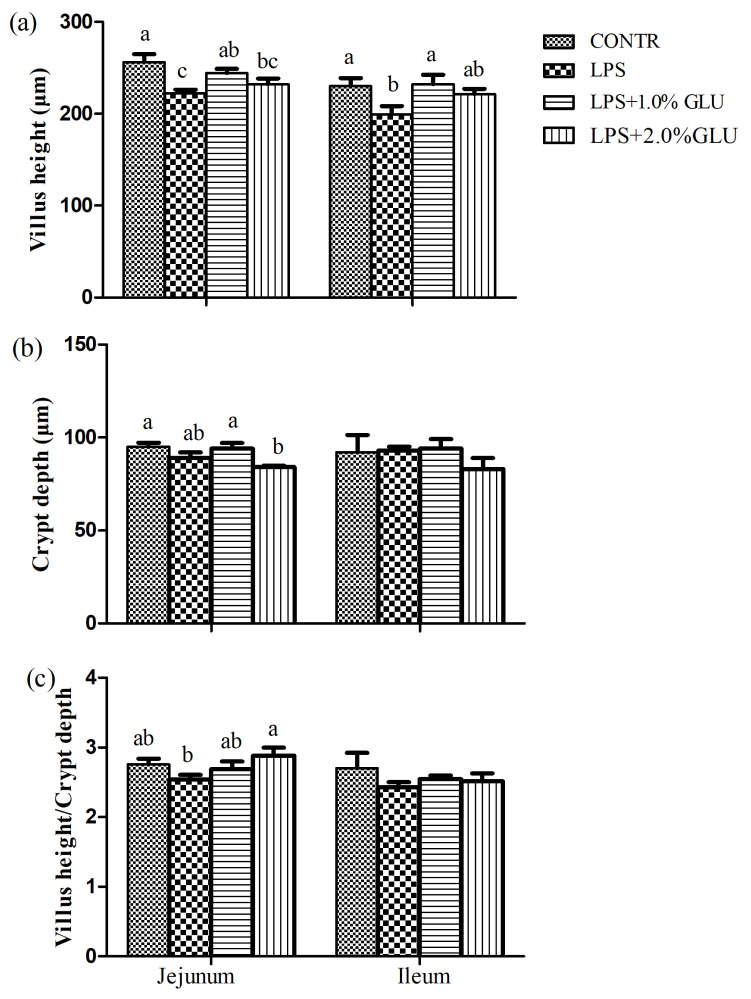
Effects of dietary glutamate (GLU) on (a) villus height, (b) crypt depth and (c) villus height:crypt depth in weaned pigs challenged by *Escherichia coli* lipopolysaccharide (LPS). Values are means and SEM, n = 6 (1 pig/pen). ( 
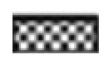
) Pigs fed a control diet and injected with sterile saline (CONTR); ( 
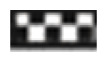
) pigs fed control diet and injected with LPS (LPS); ( 
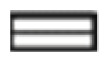
) pigs fed a 1.0%GLU diet and injected with LPS (LPS+1.0% GLU); ( 
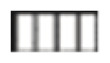
) pigs fed a 2.0% GLU diet and injected with LPS (LPS+2.0% GLU). ^a–c^ Bars with different letters indicate a significant difference (p<0.05).

Pigs challenged with LPS showed intestinal mucosal damage including shedding of epithelium at the tip of the villus and villous atrophy. GLU supplementation alleviated intestinal mucosal injury induced by LPS (Figure 2). LPS-challenged pigs had lower villus height (p = 0.003) and villus height/crypt depth (p = 0.016) in jejunum compared with pigs in CONTR group (Figure 3). Dietary supplementation with 1.0% GLU increased jejunal villus height (p = 0.035) and ileal villus height/crypt depth (p = 0.012) compared with pigs challenged with LPS. There was no significant difference in crypt depth in jejunum and ileum among all groups.

### Concentrations of amino acids in serum

Serum free amino acids analysis after 4 h LPS challenge is presented at Table 4. Compared with CONTR group, asparagine (p = 0.006), aspartic acid (p = 0.032), and tyrosine (p = 0.004) levels were lower in LPS-challenged pigs. Dietary GLU failed to restore the asparagine, aspartic acid, and tyrosine concentrations. However, pigs fed with 1.0% GLU diet had higher serum levels of arginine (p = 0.002), leucine (p = 0.014), phenylalanine (p = 0.019), methionine (p = 0.022), valine (p = 0.007), serine (p = 0.006), asparagine (p = 0.046), GLU (p = 0.014), glutamine (p = 0.011), and alanine (p = 0.017) than those in CONTR pigs.

### Ultrastructure of intestinal tight junction and tight junction protein expression

**Figure 4:**
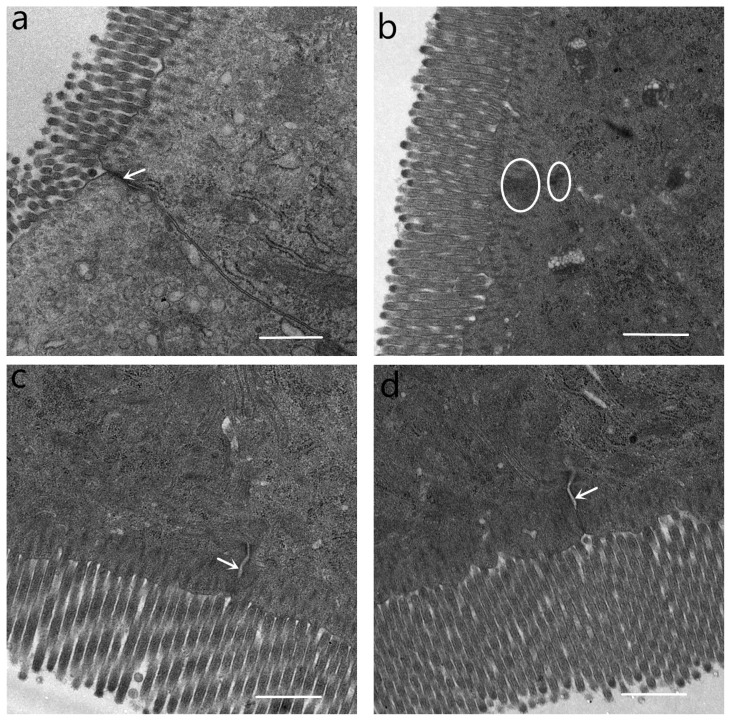
Ultrastructure of tight junction in the jejunum. (a) Pigs fed a control diet and injected with sterile saline (CONTR). Tight junction and desmosome showed an intact structure (white arrow). (b) Pigs fed control diet and injected with lipopolysaccharide (LPS). Tight junction membrane fusions were completely lost and cytoskeleton condensation (white circles). (c) Pigs fed a 1.0% glutamate (GLU) diet and injected with LPS (LPS+1.0% GLU). Tight junctions and desmosomes were intact, widening of intercellular space (white arrow). (d) Pigs fed a 2.0% GLU diet and injected with LPS (LPS+2.0% GLU). Tight junctions and desmosomes were intact, widening of intercellular space (white arrow). Original magnifications 5,000×. Scale bars = 1 μm.

Tight junctions were situated at the apical side of intestinal epithelial cell (Figure 4). At the location, tight junctions were in close proximity to the membranes, appearing to fuse at the apical side. Desmosomes were under the tight junctions. The tight junctions and desmosome displayed intact structure in CONTR pigs (Figure 4a). In contrast, tight junctions in pigs challenged with LPS were blurred. Tight junction membrane fusions were completely lost, and cytoskeleton condensation occurred. Desmosomal integrity was impaired (Figure 4b). Tight junctions and desmosomes in pigs fed with 1.0% and 2.0% GLU were intact. However, intercellular space in pigs fed with GLU widened (Figure 4c, d).

**Figure 5:**
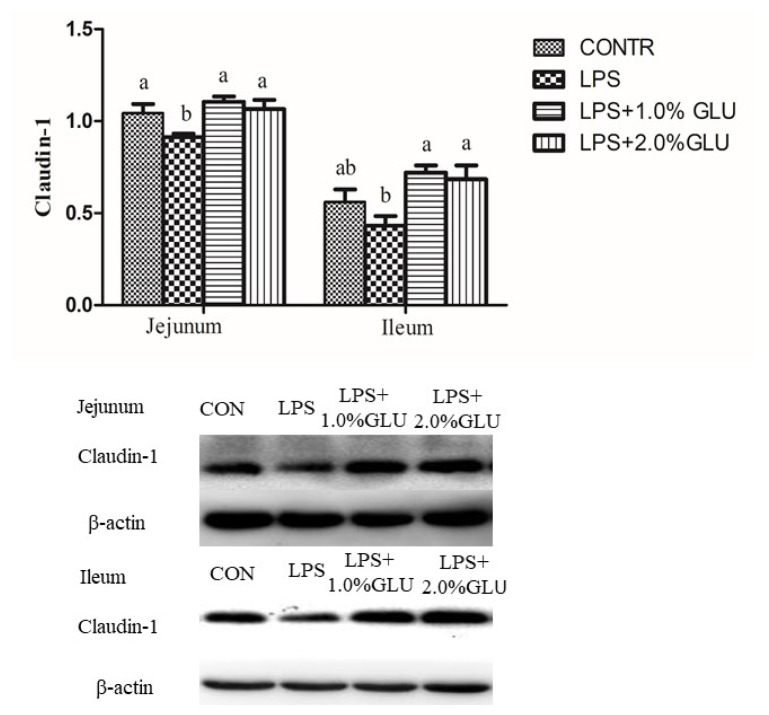
Effects of dietary glutamate (GLU) on abundance of the tight junction protein claudin-1 in weaned pigs challenged by *Escherichia coli* lipopolysaccharide (LPS). The bands are representative Western blot images of claudin-1 (22 kDa) and β-actin (42 kDa). Values are means and standard error of mean, n = 6 (1 pig/pen). ( 
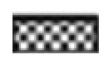
) Pigs fed a control diet and injected with sterile saline (CONTR); ( 
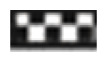
) pigs fed control diet and injected with LPS (LPS); ( 
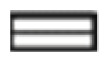
) pigs fed a 1.0% GLU diet and injected with LPS (LPS+1.0% GLU); ( 
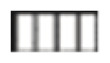
) pigs fed a 2.0% GLU diet and injected with LPS (LPS+2.0% GLU). ^a,b^ Bars with different letters indicate a significant difference (p<0.05). Data were published previously [43].

LPS challenge reduced claudin-1 expression in jejunum (p = 0.027) (Figure 5). However, pigs fed GLU had higher claudin-1 expression in jejunum (1.0% GLU, p = 0.002; 2.0% GLU, p = 0.011) and ileum (1.0% GLU, p = 0.004; 2.0% GLU, p = 0.009) compared with LPS-challenged pigs.

### Intestinal immune cells

There was no difference in the number of IEL in jejunum and ileum (Table 5) between CONTR group and LPS group. However, 1.0% GLU supplementation increased jejunal IEL number compared with LPS-challenged pigs (p = 0.012). The number of mast cells in ileum was enhanced by LPS challenge (p = 0.002), whereas dietary supplemented with GLU reduced mast cell number in ileum (1.0% GLU, p = 0.002; 2.0% GLU, p = 0.009) compared to LPS pigs. Likewise, LPS-challenged pigs had higher neutrophil number in ileum (p = 0.022) than CONTR pigs. Dietary supplementation with 2.0% GLU decreased jejunal neutrophil number (p = 0.022) compared with LPS-challenged pigs. LPS treatment and GLU supplementation had no effect on the numbers of goblet cells in jejunum and ileum.

### Serum cortisol concentration

**Figure 6:**
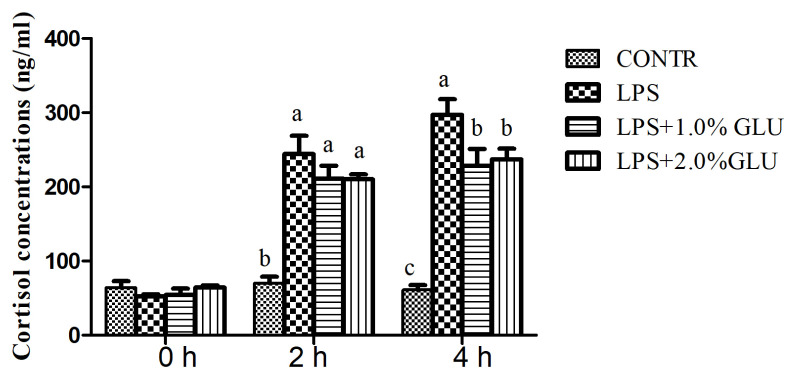
Effects of dietary glutamate (GLU) on serum cortisol concentration before (0 h), 2 h, and 4 h after *Escherichia coli* lipopolysaccharide (LPS) challenge in weaned pigs. Values are means and standard error of mean, n = 6 (1 pig/pen). ( 
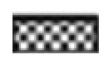
) Pigs fed a control diet and injected with sterile saline (CONTR); ( 
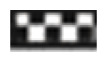
) pigs fed control diet and injected with LPS (LPS); ( 
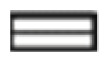
) pigs fed a 1.0% GLU diet and injected with LPS (LPS+1.0% GLU); ( 
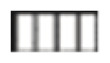
) pigs fed a 2.0% GLU diet and injected with LPS (LPS+2.0% GLU). ^a–c^ Bars with different letters indicate a significant difference (p<0.05).

Serum cortisol concentration in LPS-challenged pigs increased at 2 h (p<0.001) and 4 h (p<0.001) after LPS challenge compared with CONTR pigs (Figure 6). Pigs fed GLU diets had lower cortisol concentration at 4 h (1.0% GLU, p = 0.012; 2.0% GLU, p = 0.025) than LPS pigs.

### Intestinal mRNA expression of key genes related to CRF signaling pathway

Compared with CONTR pigs, LPS-challenged pigs had higher mRNA abundance of *CRFR1* in jejunum and ileum (p<0.001), and *CRF* (p = 0.02), *GR* (p = 0.012), and *TrKA* (p = 0.003) in jejunum (Table 6). However, dietary supplementation with GLU alleviated the increased mRNA expression of *CRFR1* in jejunum (1.0% GLU and 2.0% GLU, p<0.001) and ileum (1.0% GLU, p = 0.015; 2.0% GLU, p = 0.004), *CRF* (1.0% GLU, p = 0.008; 2.0% GLU, p = 0.016), *GR* (1.0% GLU, p = 0.013; 2.0% GLU, p = 0.001), *tryptase* (1.0% GLU, p = 0.002; 2.0% GLU, p = 0.003) and *TrKA* (2.0% GLU, p = 0.001) in jejunum. LPS challenge did not affect mRNA abundance of *NGF*, while 1.0% GLU supplementation decreased mRNA abundance of jejunal *NGF* (p = 0.01). There was no significant difference in mRNA abundance of *CRFR2* in jejunum and ileum among all groups.

### Intestinal TGFβ-1 and EGFR mRNA expression

LPS-challenged pigs had higher mRNA abundance of *TGFβ-1* (jejunum, p<0.001; ileum, p = 0.009) and *EGFR* (jejunum, p = 0.001; ileum, p = 0.027) compared with pigs in CONTR group (Table 7). Dietary supplementation of 1.0% GLU increased *TGFβ-1* mRNA abundance in jejunum (p = 0.037) compared with LPS-challenged pigs. However, 2.0% GLU supplementation decreased *EGFR* mRNA abundance in jejunum (p = 0.007) compared with LPS-challenged pigs. Furthermore, GLU supplementation did not affect mRNA abundance of *TGFβ-1* and *EGFR* in ileum compared with LPS-challenged pigs.

## DISCUSSION

GLU is the most plentiful amino acid in protein feed. It also is a multifunctional amino acid because of physiological and immune contribution. Numerous studies have demonstrated that GLU exerts various functions in nutrient metabolism, energy requirement, immune response, and oxidative stress [16,17]. Moreover, diets supplemented with GLU improved growth performance and health in pigs [18]. In this study, we established the model of stress and acute intestinal injury in weaned piglets by injecting *Escherichia coli* LPS. Previous studies demonstrated that LPS caused oxidative stress *in vivo* and *vitro* [19,20]. In fact, the antioxidant defenses including antioxidants (e.g. GSH) and antioxidant enzymes are present in the biology system [21]. As a product of lipoperoxidation, MDA is considered as the indices for oxidative stress. In the present study, LPS challenge increased MDA level and decreased GSH level, suggesting that LPS induced oxidative stress in pigs. GLU supplementation mitigated LPS induced oxidative stress by reducing MDA level and increasing GSH level. Similarly, previous study indicated that GLU and aspartate alleviated oxidative stress induced by hydrogen peroxide in weaned piglets [22]. As a precursor for GSH, GLU can increase the synthesis of GSH, which can improve intestinal redox state and protect the intestinal homeostasis.

Oxidative stress results in intestinal barrier disruption and has been linked to intestinal diseases. In the current study, we found that LPS challenge led to the decrease of villus height and villus height to crypt depth ratio, and intestinal mucosa barrier injury. Intestinal mucosa is the physical barrier against pathogens. Intestinal mucosal barrier breakdown also resulted in an increase of mucosal permeability and translocation of luminal bacteria and their products into MLN and more distant sites. Our study showed that LPS challenge enhanced translocation microorganisms in spleen and tended to increase translocation microorganisms in liver. Due to individual variation between animals, GLU supplementation only caused a numerical decrease in translocation microorganism. Moreover, GLU increased villus height and the ratio of villus height to crypt depth, alleviated the architecture injury of intestine induced by LPS challenge. Similarly, Xiao et al [23] found that GLU prevented intestinal mucosal atrophy in mouse model of total parenteral nutrition. Duan et al [24] demonstrated that dietary GLU ameliorated intestinal damage induced by mycotoxins in weaned pigs. Moreover, GLU attenuated LPS induced intestinal barrier function injury in young cyprinus carpio var [25].

Cell junction between intestinal epithelial cells is the basic structure of small intestinal barrier, which includes tight junctions, adhesion junctions and gap junctions. Among these components, the tight junctions are main factors that constitute the intestinal physical barrier. Tight junctions consist of integral membrane protein, cytoplasmic plaque protein, and cytoskeletal protein. Among integral components of tight junctions, occludin and claudin family plays an important role in regulating intestinal barrier function [26]. In line with intestinal morphology and bacterial translocation, we observed that tight junctions and desmosomal integrity were impaired in pigs challenged with LPS. GLU supplementation maintained tight junctions and desmosomal integrity and increased protein expression of claudin-1 in jejunum and ileum. Likewise, Jiao et al [27] indicated that GLU augmented expression of tight junctions and enhanced barrier function in intestinal porcine epithelial cells. In the current study, we found that dietary supplementation with GLU restored arginine, leucine, phenylalanine, valine, serine, GLU, glutamine and alanine levels. Consistently, previous studies indicated that weaning stress or mycotoxin exposure changed serum profiles of amino acid in piglets, and dietary supplementation with GLU, aspartic acid or arginine ameliorated the alteration [24,28]. GLU is substrate for the synthesis of other amino acids such as arginine, alanine. Thus, supplemental GLU could be used to synthesize these amino acids to meet physiological requirements, especially under stress condition [8,29]. Moreover, because the intestinal epithelial cells renew rapidly every few days, they have high energy demands [30]. Specifically, it requires sufficient “fuel” to preserve intestinal intactness and function under stress condition [31]. In intestine, GLU is for a large part metabolized by enterocytes for energy production [32]. Thus, GLU is the most important “fuel” for intestinal tissue. In the present study, the restoration of GLU served as major oxidative fuels in enterocytes, which was essential for intestinal cells proliferation and intestinal integrity and function.

In fact, GLU is essential to optimize the immune function of intestine and the proximal resident immune cells, including normalizing proinflammatory cytokine secretion, improving T-lymphocyte numbers, specific T cell functions, and the secretion of immunoglobulin A by lamina propria cells [33]. As expected, we observed that dietary GLU enhanced IEL and reduced neutrophil infiltration in jejunum. IEL, which reside between the cell basolateral surfaces of the epithelial, play protective effects against infection, repair of a damaged mucosal barrier, and preserving the barrier function in the intestine. So far, few studies have examined the effect of dietary GLU on IEL number in weaned pigs. Akiba et al [34] demonstrated that GLU improved mucosal defenses by preventing cellular injury in small intestine. In the present study, GLU induced IEL proliferation, which boosted damaged mucosa repair and maintained intestinal barrier integrity.

It is conceivable that stress can cause intestinal barrier dysfunction. The mechanisms of stress-related disorders involve complex brain-gut interaction. A typical-characterized physiologic response to stress is the HPA axis activation, which eventually leads to CRF and cortisol release [3]. Subsequent activated CRF and GR boosts intestinal barrier dysfunction [35]. Moreover, CRF is the significant mediator of intestinal barrier dysfunction [3]. CRF is a peptide composed of 41 amino acids, released by the central nervous system and peripheral tissues in response to stress [36]. CRF plays its roles by means of two G protein-coupled receptor subtypes: CRFR1 and CRFR2 [35]. Smith et al [35] indicated that weaning stress-induced intestinal disturbance was mediated through CRFR1 activation. In line with these findings, our study showed that LPS increased mRNA expression of *CRFR1* in jejunum and ileum, and *GR* in ileum. In addition, serum cortisol was elevated at 2 h and 4 h after LPS challenge in pigs. Meddings and Swain [37] demonstrated that the release of adrenal corticosteroids in response to stress can lead to disruption of intestinal barrier function [37]. Our results suggested that LPS challenge activated the HPA axis and *CRF* signaling pathway, and resulted in intestinal injury. Surprisingly, dietary GLU alleviated the increase of cortisol concentration and mRNA abundance of *CRF*, *CRFR1*, and *GR* caused by LPS. Similarly, Wang et al [38] demonstrated that glutamine attenuated weanling-induced increase in jejunal mRNA and protein levels of CRF. Glutamine is synthesized from GLU in nearly all cells, including intestinal epithelial cells. According to these findings, GLU is likely to suppress stress response and protect intestinal barrier function by modulating CRF signaling pathway.

Several studies have illustrated that CRF plays its roles by activating mast cell [4,35]. CRFR1 has been identified on porcine intestinal mucosal mast cells, manifesting the possibility for direct activation of mast cells by CRF [35]. Activated mast cells can proliferate, degranulate and release mediators like NGF and tryptase, which increase intestinal permeability. In turn, NGF binding its high-affinity TrkA favors mast cell proliferation and degranulation [39]. Therefore, intestinal mucosal injury and permeability further aggravate. Smith et al [35] reported that stress caused activation of mast cells and increased mast cell number [35]. Dothel et al [40] found that colonic mucosal NGF and TrkA levels were increased in irritable bowel syndrome. In line with this, our data showed that LPS increased mast cell number and mRNA abundance of *TrkA* in intestinal mucosa. GLU alleviated LPS-induced increase of mast cell number and mRNA abundance of *NGF*, *TrkA*, and *tryptase*. Therefore, GLU might prevent mast activation, resulting in a decrease of mast cell number and mRNA abundance of *tryptase* in pigs treated with LPS, and maintaining gut barrier function. The mechanism involved needs to be further investigated.

After injury, the intestinal epithelium undergoes a wound repair process, which limits inflammation and prevents future injury. Intestinal wound repair is dependent on the precise balance of migration, proliferation, and differentiation of the epithelial cells adjacent to the wounded area [4]. At the early phase of the repair, epithelial cells produce various cytokines and growth factors to modulate proliferation, migration, and differentiation of the epithelial cells. Among the modulators, TGF-β and epidermal growth factor (EGF) both act as potent modulators of intestinal epithelial proliferation [5,41]. The mammalian TGF-β family exists three isoforms: TGF-β1, -β2 and −β3. TGF-β1 is the most abundant and well-studied isoform [5]. Moreover, TGF-β1 stimulates epithelial cell migration, promotes intestinal epithelial restitution, and maintains intestinal mucosa integrity [5]. EGF also enhance epithelial cell restitution. EGF exerts the regulatory effect by binding its receptor EGFR [41]. In the current study, LPS enhanced mRNA abundance of *TGF-β1* and *EGFR* in intestinal mucosa. Similarly, Xiao et al [42] found that weaning stress caused an increase of TGF-β1 in intestinal mucosa in piglets [42]. GLU supplementation also increase mRNA abundance of *TGF-β1*. Previous studies demonstrated that GLU, as an energy source for cell proliferation, was associated with the mucosal healing process [32]. However, our results suggested that GLU might promote intestinal injury restitution which was associated with enhancing *TGF-β1* and *EGFR* expression.

## CONCLUSION

In conclusion, GLU supplementation alleviates breakdown in intestinal barrier function caused by LPS-stress. It suggests GLU maintains intestinal mucosa homeostasis, which partly is associate with regulating CRF signaling pathway.

## Figures and Tables

**Table 1:** Primer sequences used for real-time PCR

Gene	Forward (5′-3′)	Reverse (5′-3′)	Product length (bp)	Accession numbers
*CRF*	CCGCCAGGAGGCACCCGAGAGG	GCCAAACGCACCGTTTCACTTC	178	NM_001113062.1
*CRFR1*	CTCATCTCCGCCTTCATCCT	CCAAACCAGCACTTCTCATT	271	AF077185
*CRFR2*	TACAGGAAGGCGGTGAAGG	GAAGAAGCAGTAGAAGACAGACA	174	NM_001144118.1
*GR*	CCAAACTCTGCCTTGTGTGTTC	TGTGCTGTCCTTCCACTGCT	108	AY779185
*Tryptase*	ACCGGCCTGGCATCTACAC	AGGAGGTGACTGGCTTTGCA	110	AB038652
*NGF*	AGCAAGCGGTCGTCATC	CCAACACCATCACCTCCTT	124	XM_021089997.1
*TrKA*	CGGCTCACCACACTCTCAT	AGGAAGGTCACACTGGCTAAT	219	XM_021083231.1
*TGFβ-1*	CCGTCACAGAGACCACAGA	CCAGTTCAACAGGACCAAGG	222	NM_001038639.1
*EGFR*	GCTGTGGCTTGCGTTGA	CTGAGAGGCTGATTGTGGTAG	209	NM_214007.1
*GAPDH*	CGTCCCTGAGACACGATGGT	GCCTTGACTGTGCCGTGGAAT	194	AF017079.1

*CRF*, corticotrophin-releasing factor; *CRFR1*, corticotrophin-releasing factor receptor 1; *CRFR2*, corticotrophin-releasing factor receptor 1; *GR*, glucocorticoid receptors; *NGF*, nerve growth factor; *TrKA*, tyrosine kinase receptor A; *TGFβ-1*, transforming growth factor β 1; *EGFR*, epidermal growth factor receptor; *GAPDH*, glyceraldehyde-3-phosphate dehydrogenase.

**Table 2:** Effects of dietary GLU on growth performance in pigs during days 1 to 27 of experiment

Item	Treatments^1)^				SEM	Contrast^2)^		
	
	CONTR	LPS	LPS+1.0% GLU	LPS+2.0% GLU		1	2	3
ADG (g)	499	505	483	456	13	0.892	0.594	0.226
ADFI (g)	961	899	959	854	18	0.215	0.230	0.367
F/G	1.95	1.79	1.99	1.91	0.04	0.203	0.121	0.354

n = 6 (1 pig/pen).

GLU, glutamate; LPS, lipopolysaccharide; SEM, standard error of mean; ADG, average daily gain; ADFI, average daily feed intake; F/G, feed/gain.

1) CONTR, pigs fed a control diet and injected with sterile saline; LPS, pigs fed control diet and injected with lipopolysaccharide; LPS+1.0% GLU, pigs fed a 1.0% GLU diet and injected with lipopolysaccharide; LPS+2.0% GLU, pigs fed a 2.0% GLU diet and injected with lipopolysaccharide.

2) 1, CONTR vs LPS; 2, LPS vs LPS+1.0% GLU; 3, LPS+2.0% GLU.

**Table 3:** Effects of dietary GLU on translocation microorganisms of weaned pigs challenge by *Escherichia coli* LPS

Item	Treatments^1)^				SEM	Contrast^2)^		
	
	CONTR	LPS	LPS+1.0% GLU	LPS+2.0% GLU		1	2	3
MLN	4.50	5.72	4.85	4.11	0.37	0.235	0.413	0.123
Spleen	2.62	5.22	4.14	4.13	0.44	0.041	0.373	0.370
Liver	3.13	5.39	3.28	3.43	0.47	0.098	0.121	0.149

n = 6 (1 pig/pen).

SEM, standard error of mean. The values were expressed in log10 (CFU)/g of organ’s weight.

GLU, glutamate; LPS, lipopolysaccharide; CFU, colony-forming units; MLN, mesenteric lymph node.

1) CONTR, pigs fed a control diet and injected with sterile saline; LPS, pigs fed control diet and injected with lipopolysaccharide; LPS+1.0% GLU, pigs fed a 1.0% GLU diet and injected with lipopolysaccharide; LPS+2.0% GLU, pigs fed a 2.0% GLU diet and injected with lipopolysaccharide.

2) 1, CONTR vs LPS; 2, LPS vs LPS+1.0% GLU; 3, LPS+2.0% GLU.

**Table 4:** Effects of dietary GLU on serum concentration of amino acids in weaned pigs challenged by *Escherichia coli* LPS

Item	Treatments^1)^				SEM	Contrast^2)^		
	
	CONTR	LPS	LPS+1.0%GLU	LPS+2.0%GLU		1	2	3
Arginine	238	269	575	428	39	0.723	0.002	0.078
Histidine	42	33	40	42	2.30	0.193	0.376	0.203
Isoleucine	37	35	69	55	6.35	0.925	0.061	0.266
Leucine	177	181	346	227	25	0.958	0.014	0.451
Lysine	268	304	344	314	21	0.564	0.525	0.873
Phenylalanine	487	867	1,600	1,133	126	0.198	0.019	0.364
Methionine	55	45	87	90	6.70	0.558	0.022	0.015
Threonine	358	356	410	488	32	0.982	0.616	0.228
Tryptophan	56	62	75	76	7.0	0.782	0.518	0.501
Valine	107	142	278	207	20	0.436	0.007	0.167
Glycine	2,448	1,709	2,318	1,726	159	0.110	0.182	0.970
Serine	327	467	805	537	51	0.216	0.006	0.533
Tyrosine	77	31	53	44	5.9	0.004	0.106	0.365
Asparagine	51	11	35	32	4.54	0.006	0.046	0.057
Aspartic acid	46	22	43	35	3.68	0.032	0.051	0.210
Glutamate	193	242	482	383	38	0.589	0.014	0.127
Glutamine	548	567	947	536	57	0.895	0.011	0.825
Alanine	331	327	536	361	32	0.964	0.017	0.673

n = 6 (1 pig/pen).

GLU, glutamate; LPS, lipopolysaccharide; SEM, standard error of mean.

1) CONTR, pigs fed a control diet and injected with sterile saline; LPS, pigs fed control diet and injected with lipopolysaccharide; LPS+1.0% GLU, pigs fed a 1.0% GLU diet and injected with lipopolysaccharide; LPS+2.0% GLU, pigs fed a 2.0% GLU diet and injected with lipopolysaccharide.

2) 1, CONTR vs LPS; 2, LPS vs LPS+1.0% GLU; 3, LPS+2.0% GLU.

**Table 5:** Effects of dietary GLU on immune cells in the intestine of weaned pigs challenged by *Escherichia coli* LPS

Item	Treatments^1)^				SEM	Contrast^2)^		
	
	CONTR	LPS	LPS+1.0% GLU	LPS+2.0% GLU		1	2	3
Jejunum								
IEL (100 enterocytes)	19.41	19.14	21.77	19.90	0.400	0.785	0.012	0.432
Goblet cells (100 enterocytes)	4.18	5.04	4.62	4.52	0.217	0.195	0.518	0.425
Mast cells (mm^2^)	1,032	1,075	1,042	1,015	24	0.544	0.686	0.399
Neutrophils (mm^2^)	281	324	259	252	11	0.165	0.044	0.022
Ileum								
IEL (100 enterocytes)	20.85	20.51	20.42	19.96	0.327	0.731	0.925	0.613
Goblet cells (100 enterocytes)	4.09	3.45	3.52	3.38	0.217	0.222	0.895	0.900
Mast cells (mm^2^)	1,062	1,333	1,067	1,118	45	0.002	0.002	0.009
Neutrophils (mm^2^)	178	250	198	202	11	0.022	0.478	0.093

n = 6 (1 pig/pen).

GLU, glutamate; LPS, lipopolysaccharide; SEM, standard error of mean; IEL, intraepithelial lymphocytes.

1) CONTR, pigs fed a control diet and injected with sterile saline; LPS, pigs fed control diet and injected with lipopolysaccharide; LPS+1.0% GLU, pigs fed a 1.0% GLU diet and injected with lipopolysaccharide; LPS+2.0% GLU, pigs fed a 2.0% GLU diet and injected with lipopolysaccharide.

2) 1, CONTR vs LPS; 2, LPS vs LPS+1.0% GLU; 3, LPS+2.0% GLU.

**Table 6:** Effects of dietary GLU on mRNA expression (fold changes relative to CONTR) of key genes related to *CRFR* signaling pathway in the intestine of weaned pigs challenged by *Escherichia coli* LPS

Item	Treatments^1)^				SEM	Contrast^2)^		
	
	CONTR	LPS	LPS+1.0%GLU	LPS+2.0%GLU		1	2	3
Jejunum								
*CRF*	1.00	2.55	0.73	0.94	0.430	0.020	0.008	0.016
*CRFR1*	1.00	39.53	15.17	5.16	2.86	<0.001	<0.001	<0.001
*CRFR2*	1.00	0.72	0.91	0.90	0.08	0.258	0.477	0.518
*GR*	1.00	1.32	1.00	0.89	0.08	0.012	0.013	0.001
*Tryptase*	1.00	1.17	0.81	0.82	0.05	0.106	0.002	0.003
*NGF*	1.00	1.11	0.63	1.04	0.07	0.532	0.01	0.674
*TrKA*	1.00	1.67	1.45	0.93	0.09	0.003	0.281	0.001
Ileum								
*CRF*	1.00	0.99	0.94	0.68	0.17	0.963	0.857	0.243
*CRFR1*	1.00	7.65	4.30	3.57	0.98	<0.001	0.015	0.004
*CRFR2*	1.00	1.09	1.36	0.71	0.14	0.742	0.344	0.073
*GR*	1.00	0.94	1.04	1.06	0.06	0.475	0.253	0.156
*Tryptase*	1.00	0.83	0.87	0.81	0.09	0.187	0.771	0.853
*NGF*	1.00	0.65	0.55	0.79	0.09	0.105	0.659	0.509
*TrKA*	1.00	0.92	1.13	0.81	0.06	0.646	0.217	0.063

n = 6 (1 pig/pen).

GLU, glutamate; LPS, lipopolysaccharide; *CRF-R*, corticotrophin-releasing factor receptor; SEM, standard error of mean; *CRF*, corticotrophin-releasing factor; *GR*, glucocorticoid receptor; *NGF*, nerve growth factor; *TrKA*, tyrosine kinase receptor A.

1) CONTR, pigs fed a control diet and injected with sterile saline; LPS, pigs fed control diet and injected with lipopolysaccharide; LPS+1.0% GLU, pigs fed a 1.0% GLU diet and injected with lipopolysaccharide; LPS+2.0% GLU, pigs fed a 2.0% GLU diet and injected with lipopolysaccharide.

2) 1, CONTR vs LPS; 2, LPS vs LPS+1.0% GLU; 3, LPS+2.0% GLU.

**Table 7:** Effects of dietary GLU on mRNA expression (fold changes relative to CONTR) of *TGFβ-1* and *EGFR* in the intestine of weaned pigs challenged by *Escherichia coli* LPS

Item	Treatments^1)^				SEM	Contrast^2)^		
	
	CONTR	LPS	LPS+1.0% GLU	LPS+2.0% GLU		1	2	3
Jejunum								
*TGFβ-1*	1.00	1.78	2.20	1.68	0.104	<0.001	0.037	0.550
*EGFR*	1.00	1.39	1.31	1.09	0.047	0.001	0.432	0.007
Ileum								
*TGFβ-1*	1.00	1.36	1.54	1.32	0.056	0.009	0.157	0.782
*EGFR*	1.00	1.24	1.41	1.12	0.045	0.027	0.104	0.256

n = 6 (1 pig/pen).

GLU, glutamate; *TGFβ-1*, transforming growth factor β 1; *EGFR*, epidermal growth factor receptor; LPS, lipopolysaccharide; SEM, standard error of mean.

1) CONTR, pigs fed a control diet and injected with sterile saline; LPS, pigs fed control diet and injected with lipopolysaccharide; LPS+1.0% GLU, pigs fed a 1.0% GLU diet and injected with lipopolysaccharide; LPS+2.0% GLU, pigs fed a 2.0% GLU diet and injected with lipopolysaccharide.

2) 1, CONTR vs LPS; 2, LPS vs LPS+1.0% GLU; 3, LPS+2.0% GLU.
